# Multilabel SegSRGAN—A framework for parcellation and morphometry of preterm brain in MRI

**DOI:** 10.1371/journal.pone.0312822

**Published:** 2024-11-01

**Authors:** Guillaume Dollé, Gauthier Loron, Margaux Alloux, Vivien Kraus, Quentin Delannoy, Jonathan Beck, Nathalie Bednarek, François Rousseau, Nicolas Passat

**Affiliations:** 1 CNRS, LMR, UMR 9008, Université de Reims Champagne Ardenne, Reims, France; 2 CRESTIC, Université de Reims Champagne Ardenne, Reims, France; 3 Service de Médecine Néonatale et Réanimation Pédiatrique, CHU de Reims, Reims, France; 4 Unité d’aide Méthodologique - Pôle Recherche, CHU de Reims, Reims, France; 5 IMT Atlantique, LaTIM INSERM U1101, Brest, France; Western University, CANADA

## Abstract

Magnetic resonance imaging (MRI) is a powerful tool for observing and assessing the properties of brain tissue and structures. In particular, in the context of neonatal care, MR images can be used to analyze neurodevelopmental problems that may arise in premature newborns. However, the intrinsic properties of newborn MR images, combined with the high variability of MR acquisition in a clinical setting, result in complex and heterogeneous images. Segmentation methods dedicated to the processing of clinical data are essential for obtaining relevant biomarkers. In this context, the design of quality control protocols for the associated segmentation is a cornerstone for guaranteeing the accuracy and usefulness of these inferred biomarkers. In recent work, we have proposed a new method, SegSRGAN, designed for super-resolution reconstruction and segmentation of specific brain structures. In this article, we first propose an extension of SegSRGAN from binary segmentation to multi-label segmentation, leading then to a partitioning of an MR image into several labels, each corresponding to a specific brain tissue/area. Secondly, we propose a segmentation quality control protocol designed to assess the performance of the proposed method with regard to this specific parcellation task in neonatal MR imaging. In particular, we combine scores derived from expert analysis, morphometric measurements and topological properties of the structures studied. This segmentation quality control can enable clinicians to select reliable segmentations for clinical analysis, starting with correlations between perinatal risk factors, regional volumes and specific dimensions of cognitive development. Based on this protocol, we are investigating the strengths and weaknesses of SegSRGAN and its potential suitability for clinical research in the context of morphometric analysis of brain structure in preterm infants, and to potentially design new biomarkers of neurodevelopment. The proposed study focuses on MR images from the EPIRMEX dataset, collected as part of a national cohort study. In particular, this work represents a first step towards the design of 3-dimensional neonatal brain morphometry based on segmentation. The (free and open-source) code of multilabel SegSRGAN is publicly available at the following URL: https://doi.org/10.5281/zenodo.12659424.

## 1 Introduction

### 1.1 Context and objectives

Prematurity is associated with a wide range of neurological disorders, which is a significant public health concern due to the increased survival of extremely premature children [1, 2]. Prematurity exposes the brain to a number of developmental diseases, variously involving the periventricular white matter, basal ganglia, brainstem, cerebellum and maturation of cortical layers [3]. These lesions are the consequence of direct insults (inflammation directly affecting white matter) and altered maturative processes (impaired neurogenesis and synaptogenesis), collectively referred to as encephalopathy of prematurity [4]. A magnetic resonance imaging (MRI) scan of the brain at the equivalent age to term is routinely performed to identify structural lesions [5]. However, neurological disorders are not solely attributable to these obvious cerebral abnormalities [6, 7]. Preterm birth is associated with abnormal growth in many areas of the brain, associated with long-term cognitive outcomes [8, 9]. Consequently, the current interpretation of MRI acquired systematically at term equivalent age should be improved by volumetric information. Regional brain volumes are potential biomarkers that can be used to better understand the impact of prematurity on the developmental trajectory, to identify babies eligible for intervention after hospital discharge and to evaluate the effectiveness of randomised controlled trials.

Brain MRI segmentation has been explored and developed over the last two decades [10]. At present and despite an extensive literature, neonatal MRI segmentation [11] remains a research tool and has very limited application in clinical routine. In [12], we have recently proposed a new segmentation method, namely SegSRGAN, which has been specifically dedicated to the segmentation of neonatal brain MRI. SegSRGAN is based on the Generative Adversarial Networks (GAN) paradigm and aims to provide both super-resolution (SR) reconstruction of neonatal MR images (often acquired at low resolution) and segmentation of brain structures at super-resolution level. In [12], the relevance of SegSRGAN has already been proven by comparison with various state-of-the-art methods, particularly with regard to the difficult problem of segmenting the cortex.

In this article, we propose a methodological and experimental framework, based on SegSRGAN, dedicated to the parcellation and morphometric analysis of brain structures from MR images of premature infants. In particular, our contributions are threefold.

First of all, we are proposing a multi-label version of SegSRGAN. The initial version of the method, proposed in [12], could perform the binary segmentation, i.e. the extraction of one specific kind of tissue. The new multi-label SegSRGAN, proposed in this article, is now capable of performing multi-label segmentation, leading to a partitioning of the whole brain into user-selected regions of interest. The SegSRGAN multilabel (free and open-source) code is publicly available [13]. Secondly, we propose a segmentation quality control (SQC) strategy for the parcellation of brain MR images, particularly for prematurity. This SQC strategy is based on three main categories of assessment: (1) qualitative assessment by clinical experts, which aims to establish a link between the visual quality of the parcellation and the standard quality scores generally used for segmentation; (2) quantitative assessment of segmentation by comparing morphometric measurements made manually by clinical experts and automatically from segmentation; and (3) quantitative assessment of the topological accuracy of the segmentation by correlating connectivity and adjacency measurements between the segmented regions and the reference regions used to train the method. Finally, we experimentally evaluate the quality of the multi-label SegSRGAN. To this end, we consider MR images acquired in a clinical context. These images are part of a national cohort, EPIRMEX. The aim of this quality control of SegSRGAN on “real” data is to validate the approach and determine its strengths, limitations and biases, before involving it in the processing of the entire cohort for other clinical studies. x The remainder of this article is organised as follows. In the section 2, we briefly describe recent work in the different areas related to the topics of this article, namely clinical aspects of brain MRI analysis, neonatal brain segmentation and the SQC of brain MRI segmentation. In section 3.1, we present SegSRGAN. We first recall the initial, binary version of the method. We then present its extension to deal with the case of multi-label segmentation (i.e. parcellation) of the brain from MR images. We also describe a post-processing step to clean up the results, particularly with regard to extracranial artefacts. In Section 3.2, we describe our SQC protocol. We detail its three modules, which are respectively dedicated to qualitative, morphometric and topological evaluation. In section 3.3, we apply this SQC protocol to the multilabel version of SegSRGAN on a dataset constructed from the EPIRMEX cohort. We provide the numerical results of this analysis and discuss the strengths, biases and limitations of SegSRGAN in its ability to explore a full cohort of MR images.

## 2 Related works

In this section, we describe some recent contributions related to the three main issues addressed in this article: the clinical relevance of preterm brain MRI analysis (Section 2.1); recent methods for segmentation/parcellation of the neonatal brain (Section 2.2); and the development of quality control for brain MRI segmentation (Section 2.3).

### 2.1 Preterm brain MRI analysis: Clinical aspects

Over the last three decades, MRI of the neonatal brain has shown that large, obvious lesions are associated with a severe neurological course [14]. High-grade haemorrhage and parenchymal infarction are associated with cerebral palsy, low IQ and death [15]. The clinical consequences of venous infarcts (i.e. Volpe infarcts) vary according to location and size [16]. Cerebellar infarcts have a significant impact on neurological development, particularly when the vermis, or both hemispheres, are affected [17]. Cystic white matter lesions are strongly associated with cerebral palsy, but currently account for only 1% of white matter lesions. Overall, moderate and severe overt brain lesions on MRI are fairly good predictors of cerebral palsy and severe neurodevelopmental delay [18–20]. However, these obvious lesions are not the only potential consequences of premature birth on brain development. Many former preterm infants present with mild to moderate learning difficulties as well as behavioural, psychiatric and cognitive disorders [2, 21]. Brain MRI hardly predicts these mild to moderate cognitive dysfunctions by analysing only overt lesions [18].

Indeed, premature birth induces diffuse alterations in brain developmental trajectories, including structural changes in subplate, neuro-axonal organisation and cortical lamination [22]. In children born prematurely, advanced analysis of brain MRI has highlighted these structural and functional changes: gyration, structural and functional connectivity and regional volumes are altered in children born prematurely, with or without associated obvious lesions. A description of all these changes is beyond the scope of this document; readers can find more detailed information in dedicated studies [23–25].

Finally, children born prematurely show an alteration in regional brain volumes that persists throughout childhood [26, 27], and even into adulthood by drawing a morphological model of the “brain of infant born preterm” [28]. These alterations appear to correlate with neurodevelopmental prognosis [8, 29, 30]. The respective contribution of: (1) regional brain volumes [31], (2) their growth kinetics [31, 32] and (3) their asymmetry [25] for the prognosis of neurodevelopment is still controversial and under investigation. In our opinion, the biases associated with image analysis methods and their validation must be systematically taken into account, as the performance and validation of an image analysis can greatly contribute to the relevance of the biomarkers derived from its results.

### 2.2 Neonatal brain segmentation

The study of the developing brain involves several major image analysis challenges that concern the development of appropriate approaches that can cope with low contrast-to-noise ratio, rapid change in the size of brain structures, complex brightness changes in structural MRI reflecting the rapid patterning of white matter by myelination, rapid change and high variability in anatomical shapes. To address these challenges, many methods have been proposed in the literature [11, 33].

In image segmentation tasks, algorithms based on deep learning have been at the forefront of development in recent years, including in neonatal brain imaging. The U-Net architecture [34], which provides a multi-scale representation of the data, is probably the most widely used model in segmentation, particularly for neonatal data [35, 36]. We can also mention the use of other architectures such as the hyperdense-net [37], transformer weighted network [38] or attention-based networks [39].

In the context of neonatal brain imaging, deep learning segmentation algorithms are trained on large image databases, such as data from the dHCP [40] project, for which ground truth has been estimated with the DrawEM [41] method.

Deep learning methods have shown high-quality segmentation results on these research databases. However, their application on clinical data remains a challenge due to the motion artifacts present in images, the appearance variabilities of multisite data, and the anisotropic resolution of clinical data. To this end, Khalili et al. [42] have proposed a method based on generative adversarial networks (GANs) to reduce artifacts related to subject motion during acquisition. Grigorescu et al. [43] have studied two unsupervised data adaptation methods for transfer learning from one database to another. Chen et al. [44] investigated the use of GAN methods for segmentation harmonization. Finally, Delannoy et al. [12] proposed a GAN-based method for reconstructing data in highly isotropic resolution and jointly estimating a segmentation of the cortex.

In this work, we focus on the SegSRGAN method [12] to analyze anisotropic clinical data from the EPIRMEX [29] cohort associated with the EPIPAGE 2 [45] study.

### 2.3 Quality control for brain MRI segmentation

Quality control of brain segmentation is an important procedure for ensuring the relevance of a morphometric study. Automated approaches have been proposed, offering potentially reproducible and time-saving alternatives. For example, we can mention Qoala-T [46], a supervised tool for quality control of FreeSurfer segmentation maps, or MRIQC [47], which uses T1w or T2w images as input. Monereo et al. [48] recently studied the impact of these two tools for quality control and concluded that global morphological estimates such as mean cortical thickness, total surface area or estimated total intracranial volume, should be avoided to detect outliers. This study also showed that features such as Euler number could be useful for detecting inaccurate segmentation maps. Quality control of neonatal or fetal data [49–51] seems limited to image quality, with a gap in assessing the accuracy of segmentation methods. There are currently no segmentation quality control studies dedicated to neonatal brain MRI. In this work, we propose qualitative and quantitative scores to characterize segmentation maps of MR images of the neonatal brain.

## 3 Materials and methods

### 3.1 Super-resolution reconstruction and segmentation—SegSRGAN

In this work, we aim to investigate the suitability of SegSRGAN for the analysis of MR images of the neonatal brain. SegSRGAN is a hybrid method based on generative adversarial networks (GANs) [52], which aims to simultaneously perform super-resolution (SR) reconstruction and low-resolution image segmentation. Initially, SegSRGAN segmentation module was designed for binary segmentation. We first recall (Section 3.1.1) this initial method, which has been published and validated by comparison with state-of-the-art approaches in [12]. Next, we propose an extended version of SegSRGAN that is capable of multi-label segmentation, i.e. providing a parcellation of the intracranial volume into different regions. We present the modifications of this new multilabel SegSRGAN compared to the binary SegSRGAN (Section 3.1.2). As SegSRGAN is a pixel-based segmentation / parcellation approach, we also propose a post-processing procedure that aims to regularize segmentation results in a region-based paradigm, in order to eliminate semantic noise (Section 3.1.3).

#### 3.1.1 SegSRGAN: Reminder of the initial (binary) version

SegSRGAN is both an SR reconstruction method and a segmentation method. We first discuss its SR reconstruction aspect. An SR method aims at estimating a high-resolution (HR) image $X\in R^{m}$ from a low-resolution (LR) image $Y\in R^{n}$ (*m* > *n*). Such a problem can be formulated by a linear observation model:

$$\begin{array}{c} Y=H_{↓}BX+N=\Theta X+N \end{array}$$ (1)

where $N\in R^{n}$ is an additive noise, $B\in R^{m\times m}$ is a blurring matrix, $H_{↓}\in R^{n\times m}$ is a decimation matrix, and $\Theta =H_{↓}B\in R^{n\times m}$. (For low-resolution modeling, we rely on the framework proposed by Greenspan in [53], with the same parameters as in [12]).

A common way of tackling this SR problem is to define the matrix Θ^−1^ as the combination of a restoration operator $F\in R^{m\times m}$ and an interpolation operator $S^{↑}\in R^{m\times n}$ which computes the interpolated LR image $Z\in R^{m}$ associated with **Y** (i.e. **Z** = *S*^↑^**Y**). In the context of supervised learning, given a set of HR images **X**_*i*_ and their corresponding LR images **Y**_*i*_, this restoration operator *F* can be estimated such that:

$$\begin{array}{c} \hat{F}=\text{arg}\;\underset{F}{\text{min}}\;\sum_{i}d\left(X_{i}-F\left(Z_{i}\right)\right) \end{array}$$ (2)

where *d* can be, for example, a *ℓ*_2_ norm, a *ℓ*_1_ norm or a differentiable variant of *ℓ*_1_ as defined in [54].

We now focus on the segmentation part of SegSRGAN. In order to manage the trade-off between the contributions of the SR image and the segmentation in the cost function, the image segmentation problem is considered as a supervised regression problem:

$$\begin{array}{c} S_{X}=R\left(\hat{X}\right) \end{array}$$ (3)

where *R* is a non-linear function from the interpolated image $\hat{X}$ to the segmentation map **S**_**X**_. As for the SR problem, we assume that we have a set of interpolated images ${\hat{X}}_{i}$ associated with images **X**_*i*_ and their corresponding segmentation maps $S_{X_{i}}$. A general approach to solving this segmentation problem is to find the match *R* such that:

$$\begin{array}{c} \hat{R}=\text{arg}\;\underset{R}{\text{min}}\;\sum_{i}d\left(S_{X_{i}}-R\left({\hat{X}}_{i}\right)\right) \end{array}$$ (4)

GAN approaches are based on two networks. The first network, called the generator, aims to estimate, for a given interpolated input image, the corresponding HR image and segmentation map. The second network, called the discriminator, aims to differentiate “real” HR and segmentation image pairs from “generated” pairs.

*Cost function*. In order to avoid the potential problems associated with gradient saturation that can occur with the so-called “minimax” cost function usually considered in GANs, the alternative cost function WGAN-GP [55] is used. In this context, the objective is to minimize the Wasserstein distance between two distributions $P_{r}$ and $P_{g}$, corresponding here to real and generated data. Here, the discriminator learns the parameterized function *f* while the generator aims to minimize the distance. The antagonistic part of the cost function is then:

$$\begin{array}{c} L_{adv}=E_{X∼P_{X},S_{X}∼P_{S_{X}}}\left[D\left(\left(X,S_{X}\right)\right)\right]-E_{Z∼P_{Z}}\left[D\left(G(Z)\right)\right] \end{array}$$ (5)

where **X** and **S**_**X**_ are the true HR image and segmentation map, respectively, *D* is the discriminator, *G* is the generator and **Z** is the interpolated image.

Finally, the cost function to be minimized is:

$$\begin{array}{c} L_{dis}=\lambda_{gp}E_{\hat{\text{XS}}}\left[(\right\|\nabla_{\hat{\text{XS}}}D\left(\hat{\text{XS}}\right){\|}_{2}-1{)}^{2}]-L_{adv} \end{array}$$ (6)

with:

$$\begin{array}{c} \hat{\text{XS}}=\left(1-\varepsilon \right)\left(X,S_{X}\right)+\varepsilon G\left(Z\right) \end{array}$$ (7)

and *ε* ∼ *U*[0, 1], where ∇ and λ_*gp*_ > 0 are the gradient operator and its penalization coefficient, respectively.

The generator cost function is constructed by adding a pointwise comparison term *ρ* [54] between the target and the estimated images:

$$\begin{array}{c} L_{gen}=\lambda_{adv}L_{adv}+E_{X∼P_{X},S_{X}∼P_{S_{X}}}[\rho (\left(X,S_{X}\right)-G\left(Z\right))] \end{array}$$ (8)

where λ_*adv*_ > 0 is a weight that handles the trade-off between reconstruction and segmentation, and:

$$\begin{array}{c} \rho \left(\left(x_{1},\ldots ,x_{2m}\right)\right)=\frac{1}{2m}\sum_{i=1}^{2m}\sqrt{\left(x_{i}^{2}+\nu^{2}\right)} \end{array}$$ (9)

and *ν* = 10^−3^ (with *x*_*i*_ values normalized in [0, 1]). We recall that *m* is the image size. The sum in the above equation is therefore 2*m*, since *ρ* is calculated on the concatenation of segmentation and reconstruction results.

**Fig 1:**
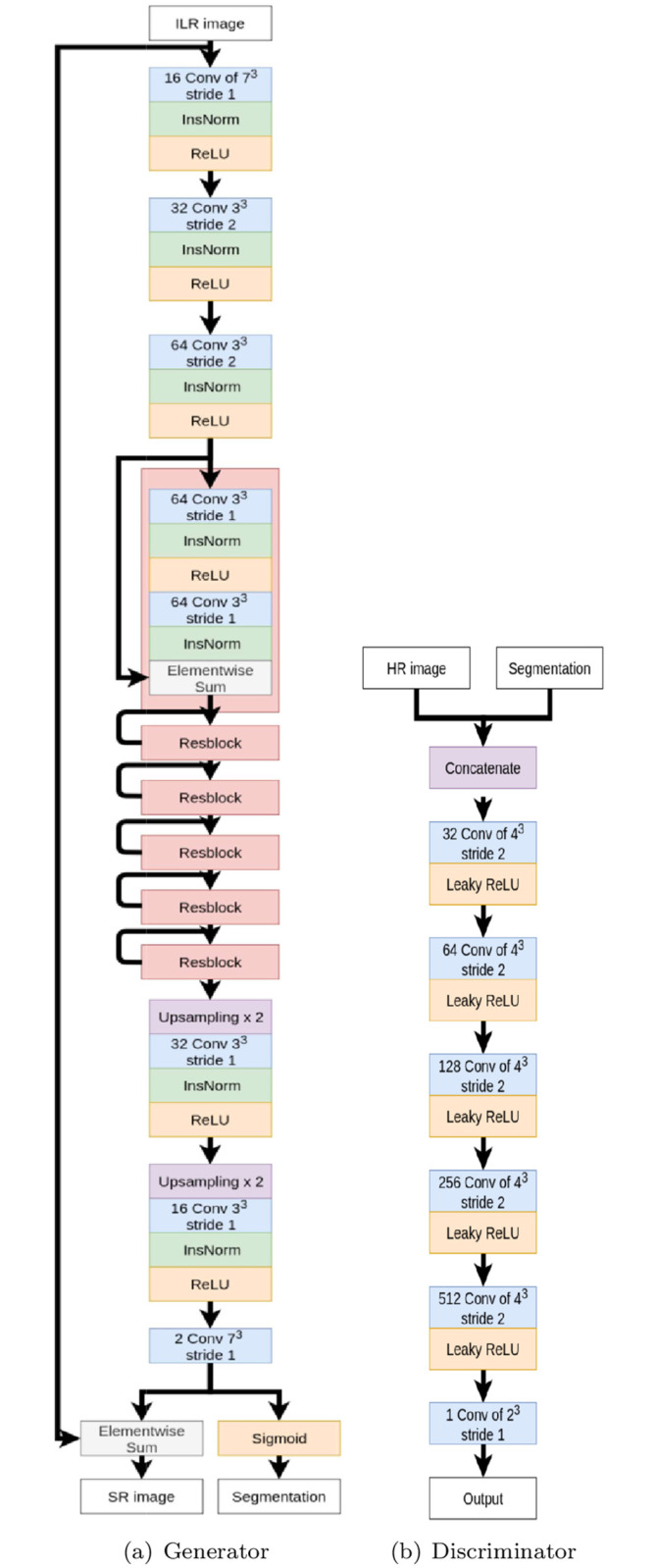
Initial SegSRGAN architecture. (a) Generator architecture. (b) Discriminator architecture.

*Network architecture*. The generator network (Fig 1a) is a convolution-based network with residual blocks. It takes the interpolated LR image as input. It comprises 18 convolutional layers: 3 for the encoding part, 12 for the residual part and 3 for the decoding part. Let $C_{j}^{i}-S^{k}$ be a block consisting of the following layers: a convolution layer of *j* filters of size *i*^3^ with stride of *k*, an instance normalization layer (InsNorm) [56] and a rectified linear unit (ReLU). *R*_*k*_ denotes a residual block as Conv-InsNorm-ReLU-Conv-InsNorm that contains 3^3^ convolution layers with *k* filters. *U*_*k*_ denotes layers as Upsampling-Conv-InsNorm-ReLU layers with *k* filters of 3^3^ and stride of 1. The generator architecture is then: $C_{16}^{7}-S^{1}$, $C_{32}^{3}-S^{2}$, $C_{64}^{3}-S^{2}$, *R*_64_, *R*_64_, *R*_64_, *R*_64_, *R*_64_, *R*_64_, *U*_32_, *U*_16_, $C_{2}^{7}-S^{1}$. During encoding, the number of cores is multiplied by 2 at each convolution, from 16 to 64. The final convolutional layer produces two 3D images: the first is transformed into a class probability map (using sigmoid activation); the second is added to the original interpolated image. To improve the performance of the learning procedure, instance normalization layers are applied to the result of each convolution (before the activation function is applied).

The discriminator network (Fig 1b) is fully convolutional. It takes as input an HR image and a segmentation map. It contains 5 convolutional layers with an increasing number of filter kernels, increasing by a factor of 2 from 32 to 512 cores. Let *C*_*k*_ be a block consisting of the following layers: a convolution layer of *k* filters of size 4^3^ with stride of 2 and a Leaky ReLU with a negative slope of 0.01. The last layer $C_{1}^{2}$ is a 2^3^ convolution filter with stride of 1. No activation layer is used after the last layer. The discriminator then consists of *C*_32_, *C*_64_, *C*_128_, *C*_256_, *C*_512_, $C_{1}^{2}$.

#### 3.1.2 Multilabel SegSRGAN

The initial SegSRGAN method described in Section 3.1.1 has been extended to segment the intracranial volume into *k* labels (*k* > 2), with the assumption that each *x*_*i*_ point of the **X** image is assigned to a unique label. This multi-label extension requires two main modifications to the initial binary version.

Firstly, the final part of the generator network dedicated to segmentation now relies on *k* convolution modules (instead of just one for the binary part). Each of these convolution modules is dedicated to a specific label, and the output of the *k* convolutions is then merged to produce the final segmentation map.

Secondly, the *ρ* error measure, which relied solely on the Charbonnier metric defined in Eq (9), now relies on two distinct metrics: Charbonnier for SR reconstruction and Dice multilabel for segmentation. The new measure *ρ*_multi_ is then defined as follows:

$$\begin{array}{c} \rho_{\text{multi}}\left(\left(X,S_{X}\right),G\left(Z\right)\right)=\rho_{\text{multi}}\left(\left(X,S_{X}\right),\left(X^{G},S_{X}^{G}\right)\right) \end{array}$$ (10)

$$\begin{array}{cc} & =\rho_{\text{Charbonnier}}\left(X-X^{G}\right)+\left(1-\rho_{\text{Dice}}\left(S_{X},S_{X}^{G}\right)\right) \end{array}$$ (11)

where *ρ*_Charbonnier_ is defined as in Eq (9) (by modifying 2*m* into *m*) and *ρ*_Dice_ is the multilabel version of the Dice measure [57]:

$$\begin{array}{c} \rho_{\text{Dice}}\left(S_{X},S_{X}^{G}\right)=\frac{2\cdot TP(S_{X},S_{X}^{G})}{2\cdot TP\left(S_{X},S_{X}^{G}\right)+FP\left(S_{X},S_{X}^{G}\right)+FN\left(S_{X},S_{X}^{G}\right)} \end{array}$$ (12)

$$\begin{array}{cc} & =2\cdot {\left(1+m/TP\left(S_{X},S_{X}^{G}\right)\right)}^{-1} \end{array}$$ (13)

with *m* the size of the image and *TP*, *FP* and *FN* the true positives, false positives, and false negatives, respectively. Note that in Eq (11), the two terms are not weighted since, by construction, they both have values in [0, 1]. The Dice loss defined in Eqs (12) and (13) is a generalization of the binary Dice loss. In practice, we assume that for any point, either the value of that point is the same in *S*_**X**_ and $S_{X}^{G}$, thus contributing to *TP*, or the value of that point is distinct in *S*_**X**_ and $S_{X}^{G}$, thus contributing symmetrically to both *FP* and *FN*. This justifies the formulation of Eq (12), where Dice’s formulation boils down to a function depending only on *TP*.

#### 3.1.3 Post-processing

The output of the segmentation process designed in SegSRGAN’s multilabel extension is a mapping *S*: Ω → *L*, where $\Omega =\left[\;\left[0,{\text{dim}}_{x}-1\right]\;\right]\times \left[\;\left[0,{\text{dim}}_{y}-1\right]\;\right]\times \left[\;\left[0,{\text{dim}}_{z}-1\right]\;\right]\subset Z^{3}$ is the MR image support and $L={\left\{\ell_{i}\right\}}_{i=0}^{k}$ is the set of labels, *ℓ*_0_ corresponding to the background (“no anatomical label”) and the other *k* *ℓ*_*i*_ each corresponding to a specific anatomical region.

The next two post-processing steps, mainly based on mathematical morphology and digital topology, aim to improve the quality of the result by eliminating artifacts and noise.

*Extracranial artifact removal*. The proposed segmentation pipeline does not include skull stripping pre-processing. Indeed, these approaches are sometimes not sufficiently robust, and can induce false negative results in the intracranial region in the event of failure. In contrast, we chose to process the entire MR image, which can lead to false positives in extracranial regions, and post-process the results to remove these artifacts later, thus securing the results in the intracranial region.

The most common artifacts are due to overestimation of external cerebrospinal fluid (CSF), which can lead to segmentation leakage during subsequent segmentation of specific extracranial structures, such as the eyes. Based on these assumptions, the post-processing proposed is as follows.

1. We construct a first volume that is the principal connected component (denoted CC(·)) of the part of Ω made up of labels that are neither background (BG) nor CSF. This first (connected) volume is denoted *T*. In particular, noting *X*_⋆_ the region of a given label ⋆, we have:
  $$\begin{array}{c} T=CC(\Omega \backslash \left(X_{\text{BG}}\cup X_{\text{CSF}}\right)) \end{array}$$ (14)
  We define a second volume *V* as the union of *T* and *X*_CSF_. We then have *V* = *T* ∪ *X*_CSF_ with *T* ∩ *X*_CSF_ = ∅.
2. Given a spherical structuring element *B*_*ρ*_ of radius *ρ*, we first apply an erosion of *V* by *B*_*ρ*_. We then retain only the largest connected component of the result. We dilate this connected component by *B*_*ρ*_ and finally find the *T* part of *V* (which must not be discarded from the result). The overall process can be seen as a connectivity-based morphological opening [58], topologically constrained by non-CSF brain tissue. It leads to the construction of a final volume *V*_*ρ*_ parameterized by *ρ*, defined as follows:
  $$\begin{array}{c} V_{\rho }=\left(\left(CC\left(V⊖B_{\rho }\right)\right)⊕B_{\rho }\right)\cup T \end{array}$$ (15)
  In particular, for any $\rho \in R_{+}$, we have:
  $$\begin{array}{c} T\subseteq V_{\rho }\subseteq V \end{array}$$ (16)
  and for any two $\rho_{1},\rho_{2}\in R_{+}$, we have:
  $$\begin{array}{c} \rho_{1}\geq \rho_{2}\Rightarrow V_{\rho_{1}}\subseteq V_{\rho_{2}} \end{array}$$ (17)
3. The definition of *V*_*ρ*_ depends on *ρ* and the optimal result may not be the same for different processed images. This optimal value $\hat{\rho }$ is determined for each image by an analysis of the elbow curve of the volume size *V*_*ρ*_.

The optimal volume $V_{\hat{\rho }}$ eliminates extracranial artifacts by assigning the label BG (non-brain tissue) to all points, i.e.:

$$\begin{array}{c} x\in \Omega \backslash V_{\hat{\rho }}\Rightarrow x\in X_{\text{BG}} \end{array}$$ (18)

*Topological noise removal*. The multi-label SegSRGAN method, like most multi-label segmentation methods, does not provide guarantees as to the topological accuracy of the results. In particular, the segmentation result may be corrupted by “label noise”, i.e. isolated voxels (or very small regions) may be mistakenly assigned a given label. In order to solve this denoising problem while avoiding, as far as possible, modifying the segmentation result provided by SegSRGAN, we propose the following post-processing, which can be considered as a multi-label version of the morphological area opening [59].

Let Π be the partition of Ω induced by the *S* segmentation and composed of the connected components of Ω for each label. Given a limit size s∈N (which can be defined as a parameter or computed by an Otsu thresholding of the histogram of the size of the connected components of the label image), our goal is to modify Π to remove all connected components *X* ∈ Π of size |*X*| < *s*. This post-processing consists of the following steps:

1. Computation of a partially labeled image *S*_0_: Ω → *L* ∪ {⊥} from *S* as follows:
  - ∀*j*, |*X*_*j*_| < *s* ⇒ ∀*x* ∈ *X*_*j*_, *S*_0_(*x*) = ⊥
  - ∀*j*, |*X*_*j*_| ≥ *s* ⇒ ∀*x* ∈ *X*_*j*_, *S*_0_(*x*) = *S*(*x*)
  We note Ω_⊥_ = {*x* ∈ Ω ∣ *S*_0_(*x*) = ⊥}.
2. Computation of a totally labeled image *S*_1_: Ω → *L* from *S*_0_ as follows:
  - ∀*x* ∈ Ω\Ω_⊥_, *S*_1_(*x*) = *S*_0_(*x*)
  - ∀*x* ∈ Ω_⊥_, *S*_1_(*x*) = *S*_0_(*y*) with $y={\text{arg}}_{\tilde{y}\in \Omega \backslash \Omega_{⊥}}\;\text{min}\;d\left(x,\tilde{y}\right)$ where *d* is the geodesic distance inside Ω_⊥_.

Step 1 is a simple operation, similar to thresholding. Step 2 can be easily implemented by an iterative process of geodesic dilations on a label image, in a framework similar to that defined in [60]. Here, the topological modeling of the image is based on the standard framework of digital topology [61], and connectedness is derived from strong adjacency (aka 6-adjacency) in $Z^{3}$.

### 3.2 Segmentation quality control protocol

Evaluating the quality of an image processing/analysis method, particularly in the context of medical image segmentation [62, 63], generally relies on calculating the usual error measures (e.g. Dice, Hausdorff distance) that assess the similarity between the results obtained and the hand-crafted annotations provided on a test dataset. In the context of segmentation of neonatal MR images, and a fortiori of premature neonates, annotations are generally not available. It is therefore reasonable to devise alternative protocols for assessing segmentation quality. In this section, we propose a segmentation quality control (SQC) protocol. It consists of three parts, which are explained as follows.

The first part of the protocol is based on the idea that a segmentation result is good if it is considered as such by experts. This part of the SQC protocol is therefore an expert-based analysis, which involves assigning scores related to specific qualitative properties that must be met by a correct segmentation result. This first part, which requires the direct participation of human experts, is described in Section 3.2.1. The second part of the protocol is based on the idea that a segmentation result is good if it enables successful subsequent analysis of the processed data. In the context of neonatal MRI, this analysis is often based on morphometric measurements (e.g. length, surface area) on image slices [64, 65]. This part of the SQC protocol, described in Section 3.2.2, requires the indirect participation of human experts, since it involves comparing morphometric measurements made by doctors directly from the images, with morphometric measurements derived from the segmentation results. The third part of the protocol is based on the idea that a segmentation result is good if it has correct intrinsic properties. These properties are linked in particular to the structure, i.e. the topology, of the segmented objects, independently of their spatial embedding. This part of the SQC protocol, described in Section 3.2.3, does not require the intervention of human experts. This involves comparing the topological properties of segmented structures with the topological properties of real structures (which, in particular, do not depend on MR images but on anatomy).

In previous work [12], we have already assessed the suitability of SegSRGAN compared to other state-of-the-art methods. Our objective here is different: to evaluate SegSRGAN’s ability to segment certain clinical data provided by clinical cohorts. It was with this in mind that we initially conceived and designed the proposed SQC protocol. In particular, in this section we describe this SQC protocol with certain parameters (e.g. number of regions) and hyperparameters (e.g. morphometric measurements, topological features) that are oriented towards our own experimental study, proposed in Section 3.3. Of course, these elements can be adapted to handle other types of images or applications that may be of interest to the reader. With this in mind, this SQC protocol should be seen as a generic, adaptable framework that offers general guidelines but no hard rules.

#### 3.2.1 Qualitative analysis

The segmentation results provided by SegSRGAN subdivide the brain into *k* regions. In our case, we set *k* = 14 (the corresponding brain regions are examined in detail in Section 3.3). Our aim in this first part of the SQC protocol is to propose a simple reading form for manually validating the segmentation results.

Here, segmentation quality is defined by the FCOO score, which is a vector score composed of four criteria: (F)rontier, (C)onnectedness, (O)verlap and (O)verflow. These criteria are detailed in Table 1. The FCOO score provides an assessment of the region’s morphology. These four criteria are complementary and determine a local anatomical score for each of the *k* specific regions. Although they are not equivalent, we can see that these four scores are in some way related to the usual quality measures, namely:

- (F)rontier: with the Hausdorff distance;
- (C)onnectedness: with the first Betti number;
- (O)verlap: with sensitivity;
- (O)verflow: with precision;

**Table 1:** Definition of the FCOO score (the higher the score, the better the quality of each criterion). See Section 3.2.1.

Criteria	Score	Evaluated features
(F)rontier	{0, 1}	Boundary of the region.
(C)onnectedness	{0, 1}	Expected number of connected components.
(O)verlap	{0, 1}	No false negatives.
(O)verflow	{0, 1}	No false positives.

An FCOO score must be provided for each labeled region of the segmentation result. This is why these scores are binary (0: incorrect; 1: correct).

#### 3.2.2 Morphometric analysis

We also want to go beyond qualitative analysis of segmented data. To obtain quantitative information, we rely on morphometric measurements generally recognized as relevant in the literature. In particular, we focus on 1-dimensional (length) and 2-dimensional (area) measurements. Basically, our aim is to quantify the extent to which these measurements made “manually” by a human expert on a native image are similar to the same measurements obtained from the binary objects given by the segmentation results.

In our study, we have taken into account some of the measures proposed in [64, 65]. In this pioneering work, measurements were made by human experts, based on their visual analysis of image slice data in the main orientations (sagittal, coronal, axial).

On the basis of this previous work, we have chosen to consider three specific measures:

- biparietal diameter (BPD);
- transcerebellar diameter (TCD);
- deep grey matter area (DGA).

The first two (BPD, TCD) are length measurements; the third (DGA) is an area measurement. In particular, the paradigm considered here is that a good segmentation is one that provides accurate morphological measurements, saving time and effort for medical practitioners.

We define below the protocol used by clinicians to provide metrics manually, considered the “ground truth”, and the protocol designed to reproduce the same metrics from segmented images.

*Manual measurements*. Each MR image is analyzed by an experienced clinician. (In our case, one expert analyzed 30 images, while a second expert analyzed 10 of these 30 images, in order to assess inter-expert agreement; the analysis was performed with 3D Slicer (https://www.slicer.org/).

**Fig 2:**
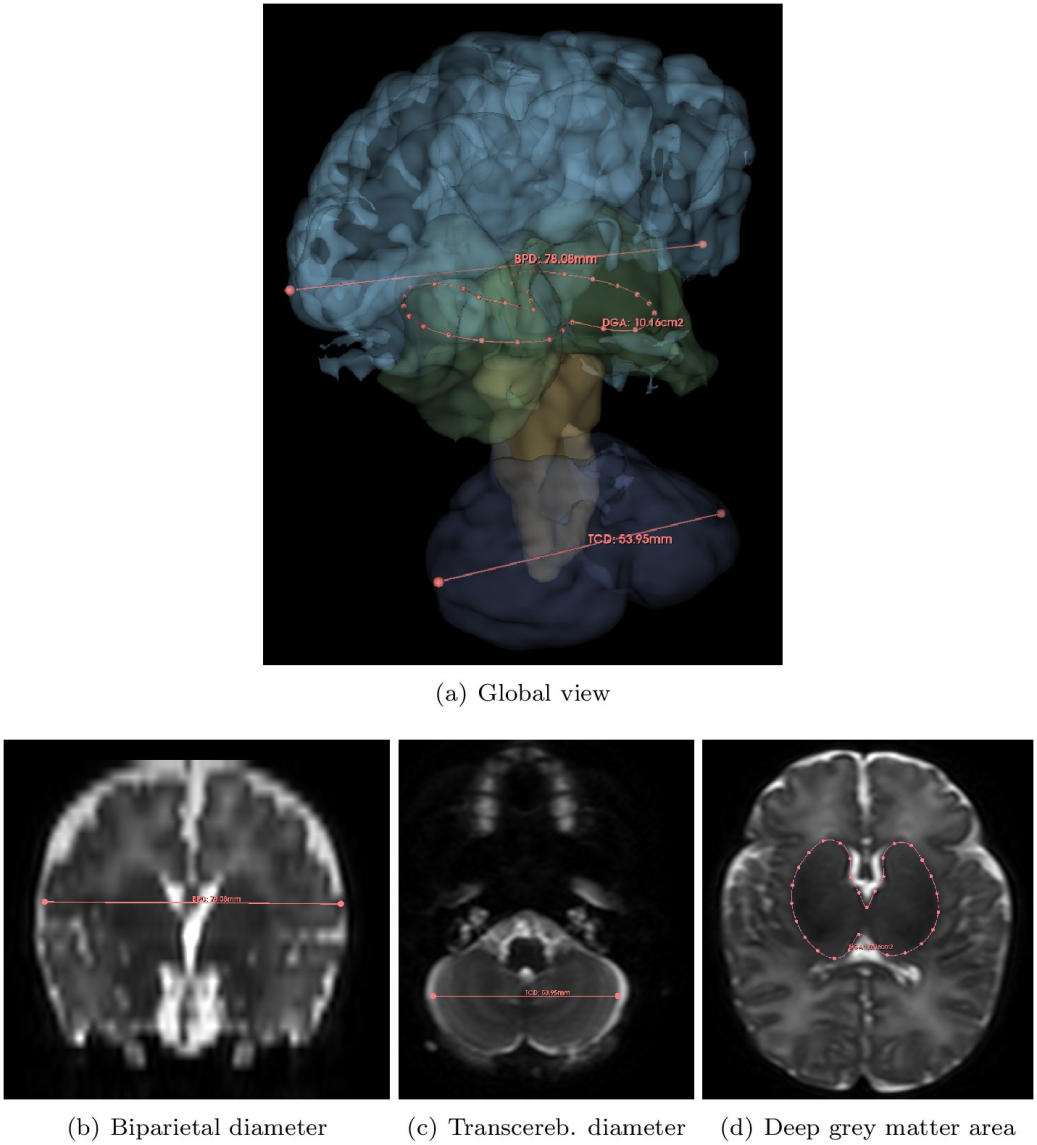
Illustration of the manual computation of the metrics. (a) 3-dimensional view of the three (length and area) measurements. (b–d) 2-dimensional view of the three measurements. (a) Biparietal diameter (BPD): the length is computed in the coronal slice. (b) Transcerebellar diameter (TCD): the length is computed in the axial slice. (c) Deep grey matter area (DGA): the area is computed in the axial slice. See Section 3.2.2.

The two length measurements (BPD, TCD) are obtained by calculating the Euclidean distance between two reference points positioned in specific coronal sections (see Fig 2). The area metric (DGA) is obtained by calculating the area of a surface defined by a spline contour generated from control points positioned within a specific axial slice.

- **Biparietal diameter (BPD)** The coronal slice is chosen as the first one located in front of the brainstem (visualized in the median sagittal slice). The beginning of the cochlea must be visible. Two points *p*_BPD_ and *q*_BPD_ are defined by the clinician. The biparietal diameter is then defined as BPD_man_ = ‖*q*_BPD_ − *p*_BPD_‖_2_.
- **Transcerebellar diameter (TCD)** The coronal slice is chosen as the one where the diameter of the cerebellum is visually assessed as maximal. The plexus can be visible and used as a reference to locate the slice. Two end points *p*_TCD_ and *q*_TCD_ are defined by the clinician. The transcerebellar diameter is then defined as TCD_man_ = ‖*q*_TCD_ − *p*_TCD_‖_2_.
- **Deep grey matter area (DGA)** The axial slice is chosen as the one where the DGA region is visually assessed as maximal. A series of points $p_{\text{DGA}}^{i}$ is set by the clinician, thus defining the contour *C*_DGA_ of a closed surface $S_{\text{DGA}}\subset R^{2}$. The area of deep grey matter is then defined as DGA_man_ = ∫∫*S*_DGA_.

*Segmentation-based measurements*. To assess the quality of the proposed segmentation, we compared these manual measurements with the measurements induced by the labeled regions. More precisely, we automatically extrapolate the morphometric measurements (length or surface) associated with the segmentation from the landmarks (control points or slice) initially determined by the human expert during his morphometric analysis. This is detailed below.

- **Biparietal diameter (BPD)** The points *p*_BPD_ and *q*_BPD_ define a line $L_{\text{BPD}}$. This line is intersected by the *R* region obtained from the label corresponding to the “Frontal No-cingulate” region, providing a segment $S_{\text{BPD}}=R\cap L_{\text{BPD}}$. The biparietal diameter estimated from segmentation is then defined as follows ${\text{BPD}}_{\text{seg}}={\left\|S_{\text{BPD}}\right\|}_{2}$.
- **Transcerebellar diameter (TCD)** The points *p*_TCD_ and *q*_TCD_ define a line $L_{\text{TCD}}$. This line is intersected by the *R*_Cer_ region corresponding to the “Cerebellum” label, giving a segment $S_{\text{TCD}}=R_{\text{Cer}}\cap L_{\text{TCD}}$. The transcerebellar diameter estimated from segmentation is then defined as ${\text{TCD}}_{\text{seg}}={\left\|S_{\text{TCD}}\right\|}_{2}$.
- **Deep grey matter area (DGA)** In the *S* axial slice chosen by the clinician, the *R*_DGA_ region corresponding to the “deep grey matter” label provides a surface ${\hat{S}}_{\text{DGA}}=S\cap R_{\text{DGA}}$ which is the segmentation analogue of the *S*_DGA_ surface defined by the clinician. The area of deep grey matter estimated from segmentation is then defined as follows ${\text{DGA}}_{\text{seg}}=\;\int \int \;S_{{\hat{S}}_{\text{DGA}}}$.

*Comparison of manual and segmentation-based measurements*. At this stage, for each of the three metrics, we have two measurements, one manual and the other based on segmentation. The error of segmentation-based measurement compared with manual measurement can be calculated absolutely and relatively as follows:

$$\begin{array}{c} \rho_{M}^{\text{abs}}=M_{\text{seg}}-M_{\text{man}} \end{array}$$ (19)

and

$$\begin{array}{c} \rho_{M}^{\text{rel}}=\frac{M_{\text{seg}}-M_{\text{man}}}{M_{\text{man}}} \end{array}$$ (20)

with M = BPD, TCD and DGA.

#### 3.2.3 Topological analysis

Discrete topology provides efficient tools for digital image analysis, particularly in the context of medical imaging [66]. In addition to the above quality scores, which are derived from ground truth and/or clinical expert analysis, i.e. extrinsic information, it is possible to design topological measures that assess the intrinsic quality of segmentation. More specifically, these topological measurements aim to quantify the accuracy of segmentation in relation to the topological properties of brain structures.

In our study, we consider a first topological metric that evaluates the connectedness of *k* labels. To this end, we define two connectedness vectors:

$$\begin{array}{c} C={\left[C_{\ell }\right]}_{\ell =1}^{k} \end{array}$$ (21)

and

$$\begin{array}{c} C\left(S\right)={\left[C_{\ell }\left(S\right)\right]}_{\ell =1}^{k} \end{array}$$ (22)

In the first, each *C*_*ℓ*_ value indicates that the region labeled *ℓ* is anatomically composed of *C*_*ℓ*_ connected components. In the second, each *C*_*ℓ*_(*S*) value indicates that the segmented region linked to the *ℓ* label is made up of *C*_*ℓ*_(*S*) connected components. For each *ℓ* label, the average error over a population of *n* patients associated with *n* segments *S*_*i*_ (1 ≤ *i* ≤ *n*) is given by:

$$\begin{array}{c} E_{C}^{\ell }=\frac{1}{n}\sum_{i=1}^{n}|C_{\ell }\left(S_{i}\right)-C_{\ell }| \end{array}$$ (23)

For the set of labels *ℓ* ∈ ⟦1, *k*⟧, the average error on a population of *n* patients associated with *n* segmentations *S*_*i*_ (1 ≤ *i* ≤ *n*) is given by:

$$\begin{array}{c} E_{C}=\frac{1}{k}\sum_{\ell =1}^{k}E_{C}^{\ell } \end{array}$$ (24)

In particular, we have $E_{C}^{\ell },E_{C}\left(S\right)\in R_{+}$ and the lower the error, the better the segmentation quality with regard to connectedness (the best score being 0).

We consider a second topological measure, linked to the adjacency relationship between the different labeling regions. Anatomically, each labeled region is adjacent to *p* other labeled regions (1 ≤ *p* ≤ *k*) and not adjacent to other *k* − *p* regions. It is then possible to design an adjacency matrix, i.e. a symmetrical square Boolean matrix *A* = (*a*_*i*,*j*_)_1≤*i*,*j*≤*k*_ where *a*_*i*,*i*_ = 1 for all labels *i* and *a*_*i*,*j*_ = 1 (resp. 0) if the regions of distinct labels *i* and *j* are adjacent (resp. non-adjacent). A segmentation map *S*, with an adjacency matrix *A*(*S*) = (*a*_*i*,*j*_(*S*))_1≤*i*,*j*≤*k*_ is defined in the same way. In this matrix, the *a*_*i*,*i*_(*S*) elements on the diagonal are set to 1 if label *i* is present in the final segmentation, and 0 otherwise. This matrix *A*(*S*) should satisfy *A* = *A*(*S*) if it is entirely correct with regard to the adjacency between the labeled regions.

For each pair of labels (*i*, *j*), the average error on a population of *n* patients associated with *n* segmentations *S*_*i*_ (1 ≤ *ℓ* ≤ *n*) is given by:

$$\begin{array}{c} E_{A}^{\left(i,j\right)}=\frac{1}{n}\sum_{\ell =1}^{n}a_{i,j}\left(S_{\ell }\right)⊕a_{i,j} \end{array}$$ (25)

where ⊕ is the “xor” operator (defined by *x* ⊕ *y* = (1 − *x*) ⋅ *y* + (1 − *y*) ⋅ *x* where *true* is associated to 1 and false to 0). For the set of label pairs (*i*, *j*) ∈ ⟦1, *k*⟧^2^, the average error on a population of *n* patients associated with *n* segmentations *S*_*i*_ (1 ≤ *i* ≤ *n*) is given by:

$$\begin{array}{c} E_{A}\left(S\right)=\frac{1}{k^{2}}\sum_{i=1}^{k}\sum_{j=1}^{k}E_{A}^{\left(i,j\right)} \end{array}$$ (26)

In particular, we have $E_{A}^{\left(i,j\right)},E_{A}^{\left(i,j\right)}\in \left[0,1\right]$ and the lower the error, the better the segmentation quality with regard to adjacency (the best score being 0).

### 3.3 Experiments

We initially designed the multilabel version of SegSRGAN (Section 3.1.2) and the SQC protocol (Section 3.2) with the aim of segmenting an entire clinical MRI cohort. In particular, our first goal was to assess the strengths and weaknesses of SegSRGAN in relation to this purpose.

#### 3.3.1 Training

*Training dataset*. The images considered for SegSRGAN training are part of the dHCP (http://www.developingconnectome.org) project [40]. The first release of the database was used. It includes infants between 37 and 44 weeks’ gestational age. T2w and inversion recovery T1w multi-slice fast spin echo anatomical images, were acquired on a 3T Philips Achieva. Infants were sleeping through the acquisition. Only T2w axial images were used for the the training set with the following characteristics: 0.8 × 0.8 mm^2^ resolution in axial planes and 1.6 mm slices overlapped.

*MRI Protocol (from* [67]*)*. The dHCP MRI protocol is documented in [67]: “*Imaging parameters were optimized for contrast to noise ratio using a Cramer Rao Lower bound approach with nominal relaxation parameter values for gray matter T1/T2*: 1800/150 *ms and white matter T1/T2*: 2500/250 *ms. T2w and inversion recovery T1w multi-slice FSE images were each acquired in sagittal and axial slice stacks with in-plane resolution* 0.8 × 0.8 *mm*^2^ *and* 1.6 *mm slices overlapped by* 0.8 *mm (except in T1w Sagittal which used a slice overlap of* 0.74 *mm). Other parameters were–T2w: TR/TE* = 12000/156 *ms, SENSE factor* 2.11 *(axial) and* 2.60 *(sagittal); T1w: TR/TI/TE* = 4795/1740/8.7 *ms, SENSE factor* 2.27 *(axial) and* 2.66 *(sagittal). 3D MPRAGE images were acquired with* 0.8 *mm isotropic resolution and parameters: TR/TI/TE* = 11/1400/4.6 *ms, SENSE factor* 1.2 *RL (Right-Left). The FSE acquisitions were each reconstructed using a motion correction algorithm and then the transverse and sagittal images were fused into a single 3D volume for each modality using slice-to-volume methods*.”

dHCP provides a parcellation of the brain into 87 labels/classes (https://gin.g-node.org/BioMedIA/dhcp-volumetric-atlas-groupwise/raw/master/config/structures.txt). We chose to reduce the number of classes from 87 to 14 in order to train SegSRGAN. Designing these new labels simply involved grouping the original 87 labels into 14 subsets, as defined in Table 2. In our case, the choices leading to this grouping were motivated by a more in-depth analysis of specific regions that could be used to define biomarkers. In other words, the definition of labels (number and type) is a meta-parameter that depends on the clinical objective. In particular, any other grouping can be considered, including the preservation of the original 87 labels. It should be noted that each new grouping may require specific training. An exception can be made where a second grouping refines a first. In this case, the second learning can be initialized with the results of the first, following a fine-tuning paradigm. In practice, basal ganglia labels were grouped together, along with ventricular system labels. Gray matter and white matter labels from the same lobe were grouped together as we observed a volume interdependence between these two areas depending on imaging quality and degree of myelination. Moreover, from a physiological point of view, the cortex is connected to the underlying white matter, which contains axons originating from cell bodies located in the cortex. Finally, in the premature brain, subcortical white matter is occupied by the subplate, which is intimately linked to the cortex. Assuming that a median cutting plane would enable the right and left parts of each volume to be individualized, we grouped the right and left sides of each volume together. Finally, we retained a higher level of temporal lobe segmentation to distinguish auditory and language centers, whose functional maturation is central in premature infants and is the subject of much research [28]. This finally led us to define the 14 macroscopic regions of interest detailed in Table 2. Note that dHCP also provides a label grouping of the same order as that proposed here. The proposed SegSRGAN multilabel implementation can handle any partition of arbitrary size, allowing interested users to experiment according to their own objectives.

**Table 2:** The 14 labels corresponding to the considered anatomical regions (and their correspondence with the 87 dHCP label identifiers). See Fig 3.

Id	Label	Anatomical region	dHCP Identifiers
1	A	Occipital	22–23, 65–66
2	B	Parietal	38–39, 81–82
3	C	Cerebellum	17–18
4	D	Corpus callosum	48
5	E	Brainstem	19
6	F	Deep grey matter	40–47, 85–87
7	G	Frontal no-cingulate	36–37, 79–80
8	H	Frontal cingulate	32–35, 75–78
9	I	Temporal auditory	11–12, 30–31, 57–58, 73–74
10	J	Temporal insula	20–21, 63–64
11	K	Temporal internal	1–6, 9–10, 15–16, 24–27, 51–52, 55–56, 61–62, 67–70
12	L	Temporal lateral	7–8, 13–14, 28–29, 53–54, 59–60, 71–72
13	M	Ventricle lateral	49–50
14	N	Cerebral spinal fluid	83

**Fig 3:**
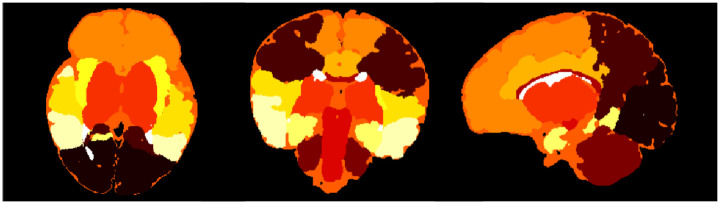
Example of the 14-label map obtained from the the 87-label map of dHCP image. Each colour corresponds to a distinct label. Axial, coronal and sagittal cross-section views.

A visual representation of the induced label map is shown in Fig 3. Note that cerebrospinal fluid is one of the 14 regions. In practice, the segmentation of this region, which plays to some extent the role of “background” in the intracranial volume, was not evaluated in our SQC protocol.

*Training SegSRGAN*. Segmentation training was performed on compute nodes at the ROMEO regional computing center (https://romeo.univ-reims.fr) with the following 2018 supercomputer parameters: a compute node is composed of 2× Intel Xeon Gold “Skylake” 6132 (2 × 14 core 2.60 GHz), 4× NVidia Tesla P100/16GB SXM2 GPU. Available memory consists of 12 times 8@2667MT/s DDR4 DIMMs (96GB per node) and 2× Bull BXI connection for interconnection.

For the current study, different sets of parameters were tested to form the GAN architecture (see Fig 1). Based on this analysis, we have chosen a batch size of 27 and 300 epoch iterations. For the images, training was based on a stride of 20, a 128 patch size and a step 20 between patches. Regarding the discriminator loss $L_{dis}$ (see Eq (6)), we chose λ_*gp*_ = 1 ⋅ 10^2^. Regarding the generator loss $L_{gen}$ (see Eq (8)), we set λ_*adv*_ = 1 ⋅ 10^−3^. We set a learning rate of 1 ⋅ 10^−4^ for both networks. Training was performed on a set of 32 images of the dHCP dataset. Testing was carried out on a set of 8 images from the dHCP dataset. We assessed the accuracy of segmentation with respect to each label based on the Dice score. These Dice scores (mean ± standard deviation) are given in Table 3. The numbers of the labels refer to the labels as defined in Table 2. These results are satisfactory, with Dice values from 0.811 to 0.949.

**Table 3:** Dice score ± standard deviation of segmentation results for each label.

1	2	3	4	5	6	7
0.920	0.925	0.946	0.811	0.949	0.879	0.937
±0.012	±0.010	±0.014	±0.025	±0.006	±0.090	±0.020
8	9	10	11	12	13	
0.871	0.849	0.890	0.893	0.886	0.871	
±0.010	±0.031	±0.035	±0.014	±0.018	±0.020	

#### 3.3.2 Data

*EPIRMEX cohort*. The images considered in this study are part of the EPIRMEX dataset. EPIRMEX is a French research project aimed at establishing a correlation between brain MRI at birth and cognitive outcomes in extremely premature infants. This is an ancillary study of the EPIPAGE-2 project (https://epipage2.inserm.fr) [68], which recruited 5170 children born before 32 weeks’ gestation and collected demographic, clinical and follow-up data up to 12 years. In the EPIRMEX subset, 581 children from 12 hospitals were recruited from June 30, 2011 (the study ended on December 21, 2017) and underwent brain MRI at term equivalent age (TEA-MRI). Neonatologists specializing in newborn brain MRI interpretation participated in the centralized expert review of these data. In addition, DICOM files of the images were collected for image processing purposes.

*EPIRMEX MRI protocol (from* [29]*)*. The EPIRMEX MRI protocol is documented in [29]: *“MRI brain scans were performed in natural sleep at TEA (i.e., GA of 39-41 weeks), using a 1.5T or 3T MRI system with a dedicated 8-channel head coil. MR devices with a magnetic field of 1.5T were Philips Achieva, Philips Intera, Toshiba MRT 200, GE SignaHdxt (General Electric Healthcare), Siemens Avanto, Siemens Symphony, and Siemens SymphonyTim (Siemens Heathineers). The MRI device with a magnetic field of 3T was a Philips Achieva (Philips Healthcare). T2 datasets were obtained using an axial T2 morphological sequence (fast spin echo/turbo spin echo with a 90 flip-back pulse); slice thickness, 3 mm; pixel size*, 0.39 × 0.39 *mm*^2^; *field of view*, 192 *mm; repetition time*, 6680 *ms; echo time*, 142 *ms; flip angle*, 120°. *The axial MRI reference plane was the bi-commissural plane. […] A medical engineer visited all participating centers to check the sequence parameters. The infants were fed, swaddled and had earplugs. No child has received medicated sedation. Throughout the scan, infants were monitored using an apnea monitor and an oxygen saturation probe, and if required, oral sucrose was administered with parental consent.”*

*Choice of a subset of data*. We only worked on images acquired at a single hospital center, as the characteristics and settings of the MRI at each center may affect segmentation. Furthermore, we only analyzed images acquired with a TE of 280 ms. Indeed, the most visually satisfying results were obtained around 280 ms, which we have kept for future use. The subset of data from center A, which contained the largest number of MR images at 280 ms, was therefore retained.

### 3.4 Ethics statement

dHCP data is publicly available (https://biomedia.github.io/dHCP-release-notes/index.html), so its use does not require the approval of a local ethics committee. In particular it is stated in [67] that *“The studies involving human participants were reviewed and approved by United Kingdom Health Research Authority (Research Ethics Committee reference number: 14/LO/1169). Written informed consent to participate in this study was provided by the participants’ legal guardian/next of kin.”*.

EPIPAGE-2 study was approved by the national data protection authority (*Commission Nationale de l’Informatique et des Libertés*, CNIL n°911009) and by the appropriate ethics committees, i.e. the advisory committee on the treatment of personal health data for research purposes (CCTIRS: *Comité Consultatif sur le Traitement de l’Information en matière de Recherche*, approval granted November 18, 2010; reference number 10.626) and the committee for the protection of people participating in biomedical research (CPP: *Comité de Protection des Personnes*, approval granted March 18, 2011, reference CPP SC-2873); see [45].

## 4 Results

**Fig 4:**
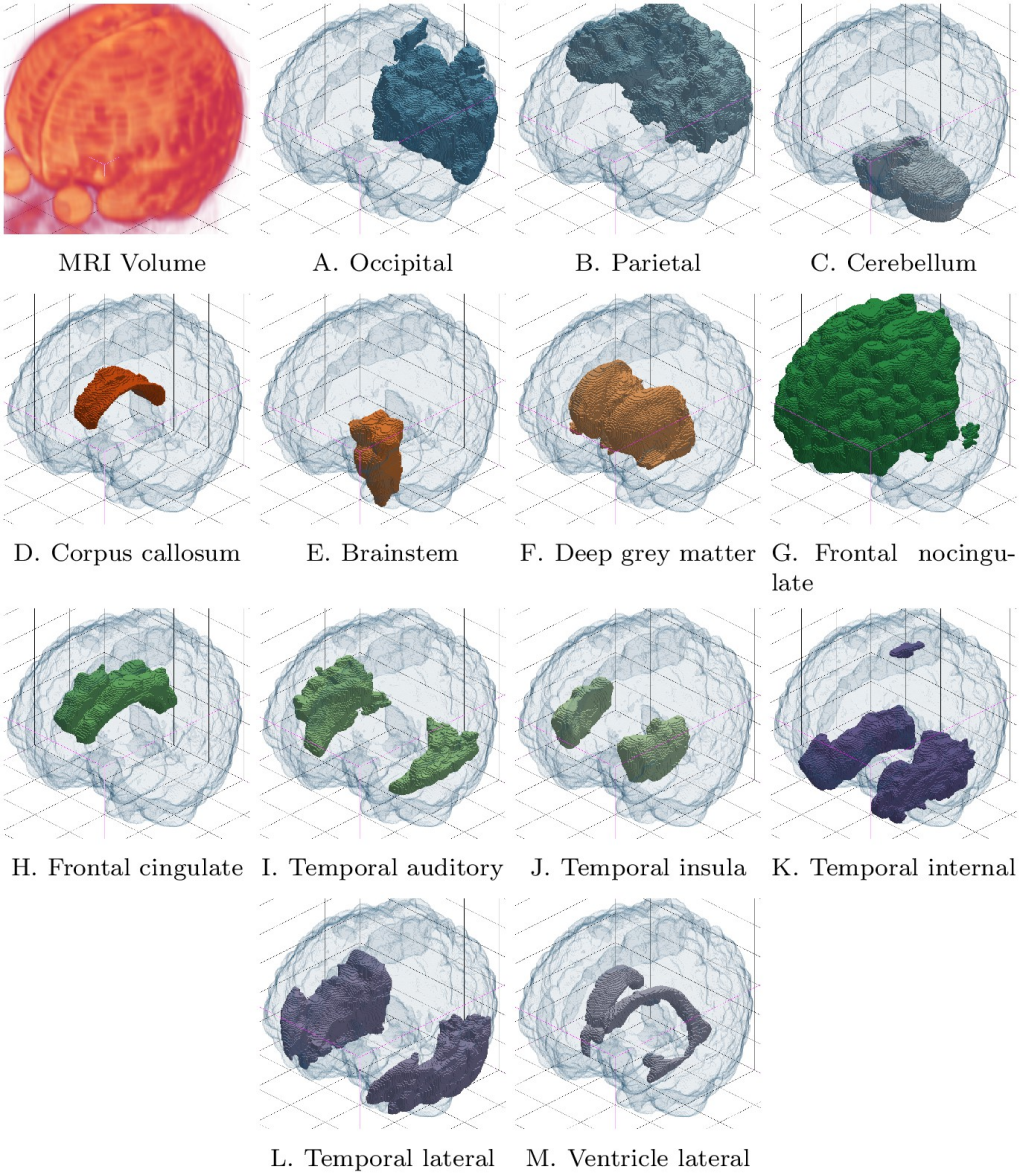
Segmentation result (labels A–M, see Table 2) on one MR image of the dataset For the sake of visualization, each of the labels is represented standalone, as a binary segmentation map.

The EPIRMEX subset composed of the 70 images described in Section 3.3.2 was processed by SegSRGAN. A segmentation result for one of these images is shown in Fig 4, by way of illustration. These segmentation maps were used for the SQC protocol described in Section 3.2. The use of EPIRMEX images allows us to evaluate the relevance of SegSRGAN on real clinical data.

### 4.1 Segmentation quality control—Part 1: Qualitative analysis

**Fig 5:**
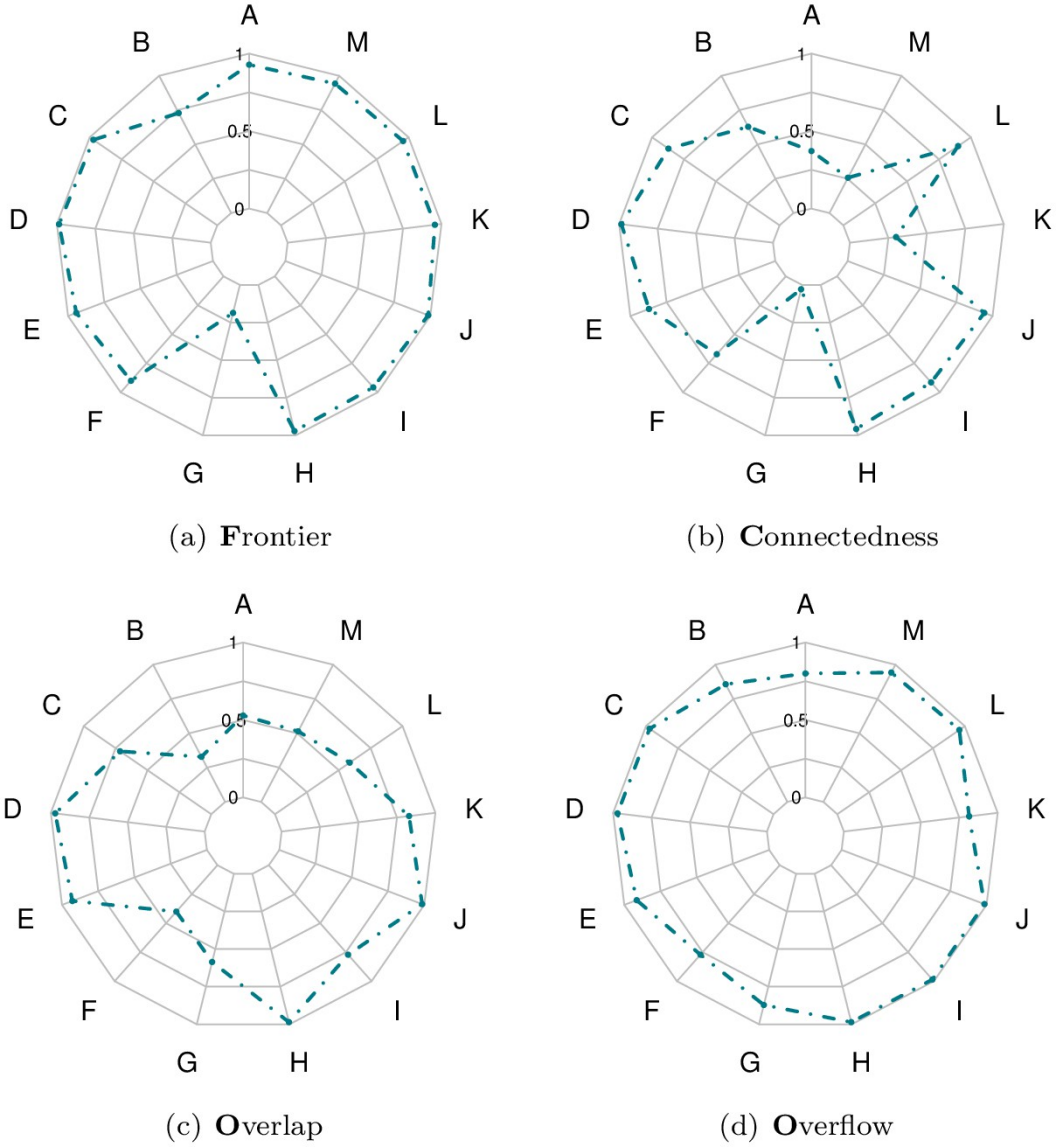
Kiviat diagrams for the qualitative analysis of the SQC protocol: (a) Frontier; (b) Connectedness; (c) Overlap; (d) Overflow. Each point of a diagram corresponds to a mean score in [0, 1] obtained as the mean value over the tested segmentations (See Tables 1 and 2).

**Fig 6:**
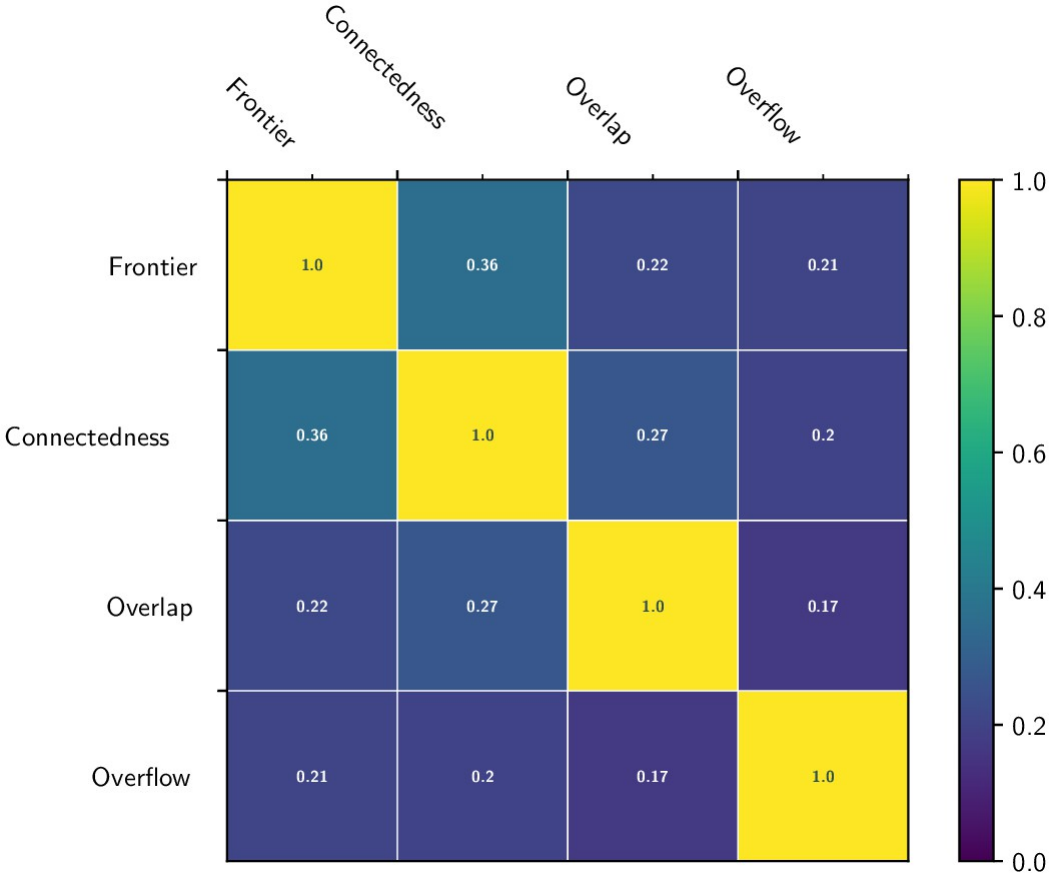
Correlation (symmetric) matrix between the four FCOO scores.

As indicated in Section 3.2.1, the first part of the SQC protocol is based on a qualitative analysis formalized by FCOO scores for each of the 13 labeled regions. For each score, namely (F)rontier, (C)onnectedness, (O)verlap, (O)verflow, and for each label, the mean value over all 70 patients was calculated. The results are summarized in the four Kiviat diagrams described in Fig 5 (one diagram per score). These diagrams are oriented from 0 (diagram center) to 1 (diagram border). The closer this boundary is to 1, the better the value of the average score for a given score and a given label. The correlation between the four FCOO scores is shown in Fig 6.

### 4.2 Segmentation quality control—Part 2: Morphometric analysis

**Fig 7:**
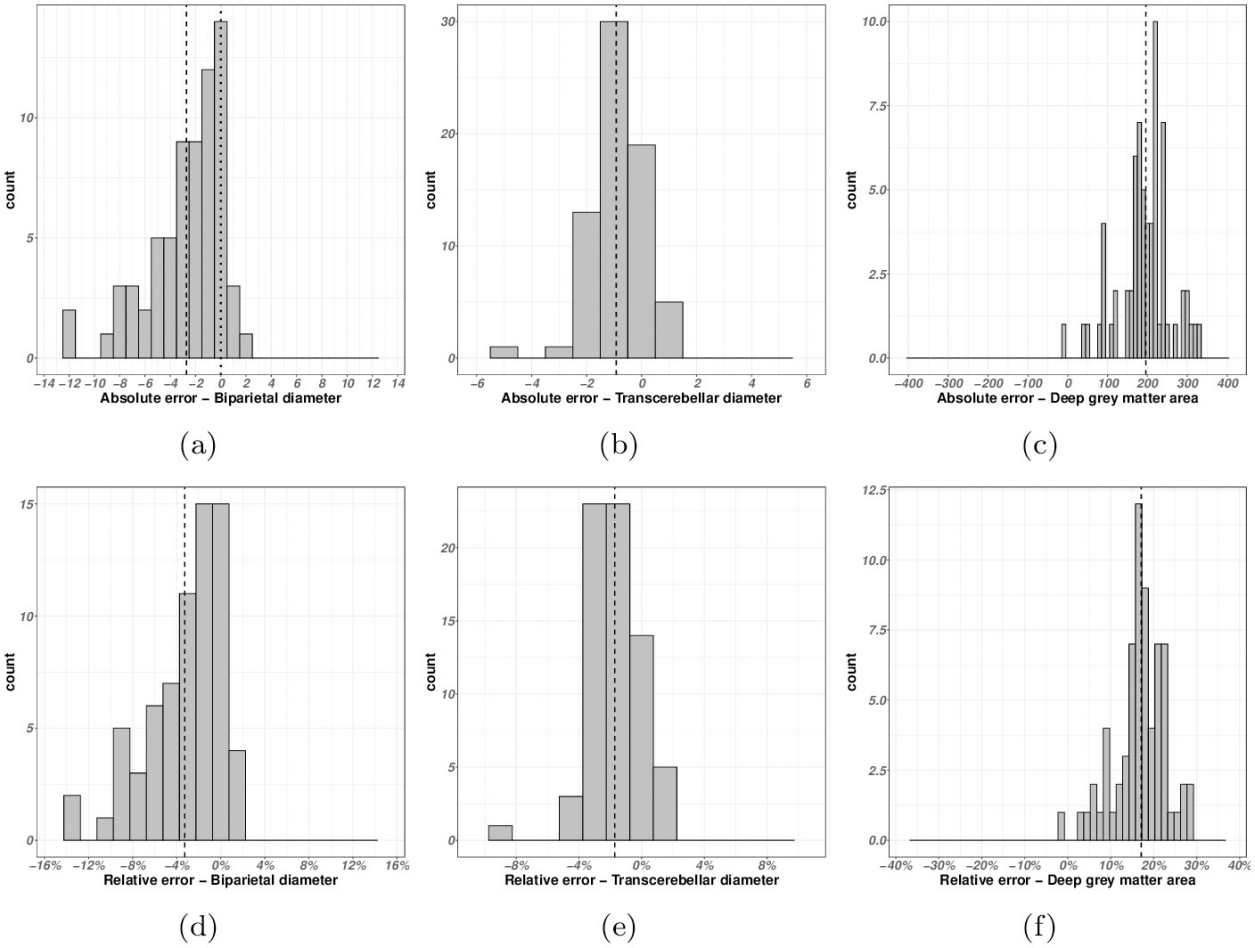
Histograms of the errors between hand-made morphometric measures and segmentation-guided morphometric measures (see Sections 3.2.2 and 4.2). (a–c) Absolute errors. (d–f) Relative errors. (a,d) Biparietal diameter (BPD). (b,e) Transcerebellar diameter (TCD). (c,f) Deep grey matter area (DGA). For the sake of visualization, the number of bins has been optimized with respect to the distributions. The vertical dashed line corresponds to the average error.

As indicated in Section 3.2.2, the morphometric analysis part of the proposed SQC protocol can be performed by calculating the error between manual measurements (length, area) of certain structures of interest obtained from native images, and the same measurements obtained from segmentation of these structures. We focus here on three measures: biparietal diameter (BPD), transcerebellar diameter (TCD) and deep gray matter area (DGA). For each, 30 patients from the dataset were involved. The absolute and relative errors obtained from these experiments are summarized by the histograms in Fig 7.

### 4.3 Segmentation quality control—Part 3: Topological analysis

In order to assess the quality of the segmentation results with regard to connectedness and adjacency, it is mandatory to determine the ground truth for these two features, i.e. to define the connectedness vector *C* (Eq (21)) and the adjacency matrix *A*. In particular, we define the connectedness vector as follows:

$$\begin{array}{c} C={\left[C_{\ell }\right]}_{\ell =1}^{13}=\left[1,1,1,1,1,1,1,1,2,2,2,2,1\right] \end{array}$$ (27)

From an anatomical point of view, each labeled region is connected, i.e. composed of one connected component, with the exception of regions whose parts are symmetrical (left and right), which are composed of two connected components. Labeled regions in a segmented image should have the same connectivity properties.

Given a label *ℓ*, we denote *C*_*ℓ*_(*S*) the number of connected components of the label region *ℓ* in the segmentation map *S*. A segmentation *S* correct with regard to connectedness should then have a vector $C\left(S\right)={\left[C_{\ell }\left(S\right)\right]}_{\ell =1}^{k}$ equal to the vector *C* (see Eq (22)).

**Fig 8:**
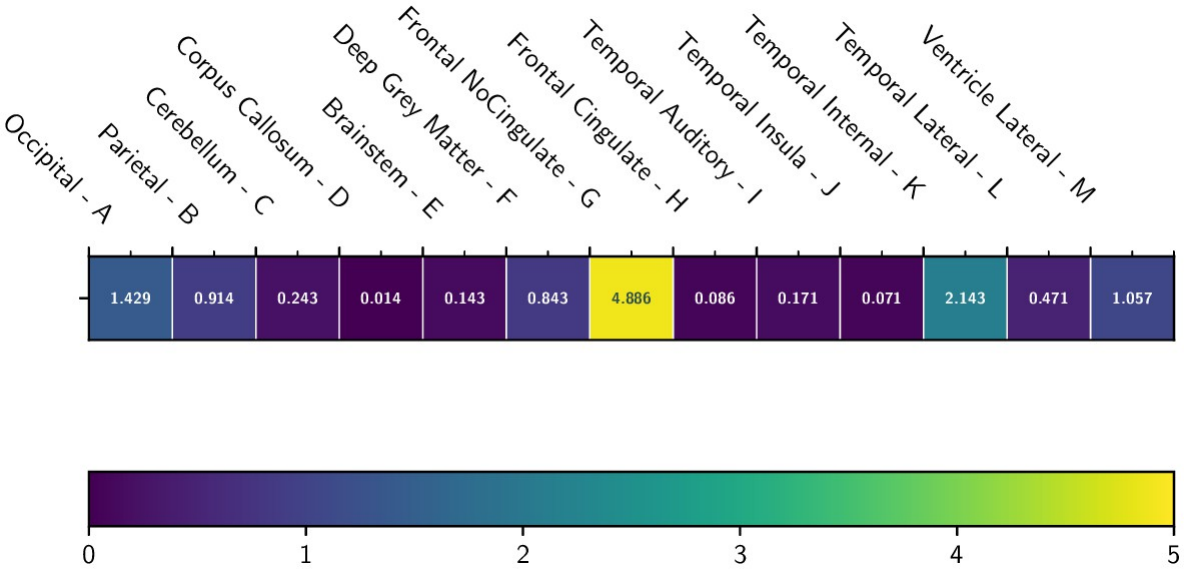
Average connectedness error $E_{C}^{\ell }\left(S\right)$ for each of the 13 labels *ℓ*, computed over 70 images, with a heatmap coloration.

The overall quality of the *S* segmentation with respect to the connectedness feature is then given by the global and label-level error measures $E_{C}^{\ell }$ and $E_{C}$ defined in Eqs (23) and (24), respectively. Here, the global error is $E_{C}=0.9593$. The 13 error measurements per label $E_{C}^{\ell }$ are shown in Fig 8.

For the measurement of adjacency error, we set the adjacency matrix as induced by the ground truth of dHCP:

$$\begin{array}{c} A={\left(a_{i,j}\right)}_{1\leq i,j\leq 13}=\left\{\begin{array}{ccccccccccccc} 1 & 1 & 1 & 0 & 0 & 0 & 0 & 0 & 0 & 0 & 1 & 1 & 1\\ 1 & 1 & 0 & 1 & 0 & 1 & 1 & 1 & 1 & 1 & 1 & 1 & 1\\ 1 & 0 & 1 & 0 & 1 & 0 & 0 & 0 & 0 & 0 & 1 & 1 & 0\\ 0 & 1 & 0 & 1 & 0 & 1 & 1 & 1 & 0 & 0 & 0 & 0 & 1\\ 0 & 0 & 1 & 0 & 1 & 1 & 0 & 0 & 0 & 0 & 1 & 0 & 0\\ 0 & 1 & 0 & 1 & 1 & 1 & 0 & 1 & 1 & 1 & 1 & 1 & 1\\ 0 & 1 & 0 & 1 & 0 & 0 & 1 & 1 & 0 & 0 & 0 & 0 & 1\\ 0 & 1 & 0 & 1 & 0 & 1 & 1 & 1 & 1 & 1 & 0 & 0 & 1\\ 0 & 1 & 0 & 0 & 0 & 1 & 0 & 1 & 1 & 1 & 1 & 1 & 1\\ 0 & 1 & 0 & 0 & 0 & 1 & 0 & 1 & 1 & 1 & 1 & 1 & 1\\ 1 & 1 & 1 & 0 & 1 & 1 & 0 & 0 & 1 & 1 & 1 & 1 & 1\\ 1 & 1 & 1 & 0 & 0 & 1 & 0 & 0 & 1 & 1 & 1 & 1 & 1\\ 1 & 1 & 0 & 1 & 0 & 1 & 1 & 1 & 1 & 1 & 1 & 1 & 1 \end{array}\right\} \end{array}$$ (28)

**Fig 9:**
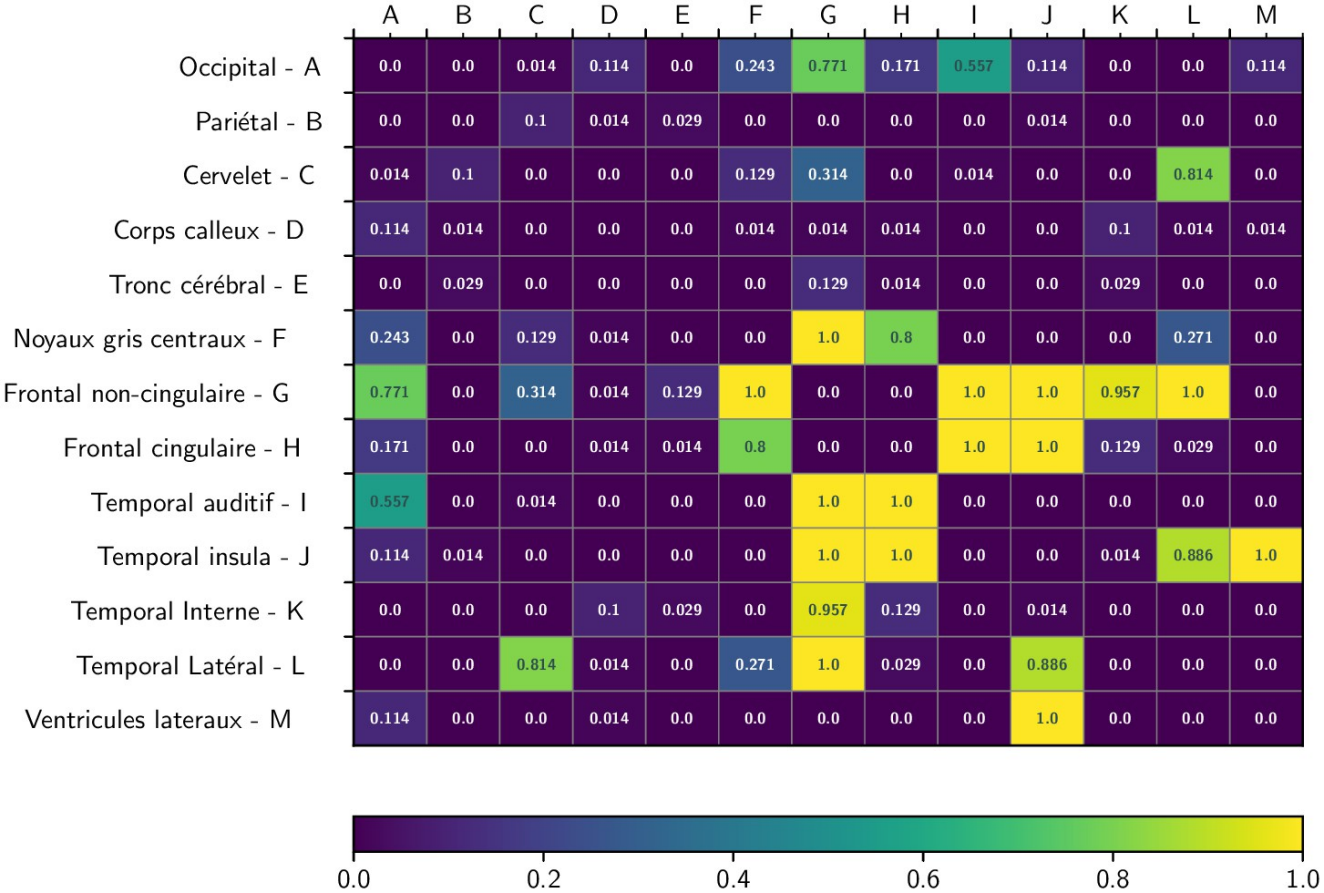
Average adjacency error $E_{A}^{\ell }\left(S\right)$ for each of the pairs of labels, computed over 70 images, with a heatmap coloration.

The overall quality of the *S* segmentation with respect to the adjacency feature is then given by the pairwise and global error measures $E_{A}^{i,j}$ and $E_{A}$ defined in Eqs (25) and (26), respectively. Here, the global error is $E_{A}=0.1534$. The 91 error measurements per label $E_{A}^{i,j}$ are represented in the (symmetrical) matrix in Fig 9.

## 5 Discussion

In this section, we examine the results presented in Section 4, from both a methodological and clinical point of view.

Firstly, the qualitative results illustrated in Fig 4 highlight the ability to correctly segment structures and tissues with salient contours. The Kiviat diagrams presented in Fig 5, which summarize the results obtained by the experts, confirm the robustness of the method in terms of segmented region boundary accuracy. In fact, for 12*ofthe*13 regions, the associated scores are very good. Again based on the Kiviat diagrams, the quality of the overflow also appears to be very good. On the other hand, connectedness and overlap seem less consistent, with some regions showing excellent results, while others are less satisfactory. As for the correlation between these scores, summarized in Fig 6, we observe a low pairwise correlation for the four FCOO scores (0.17 to 0.36). This tends to confirm the relevance of considering these 4, complementary scores.

As far as morphological scores are concerned, we observe little dispersion of error between segmentation-based and expertise-based measures. This error varies from −10% to + 2% for the biparietal diameter and from −6% to + 2% for the transcerebellar diameter in relation to the histogram maximum. It varies from 0% to + 30% for the deep gray matter zone. This confirms the ability of a segmentation-based morphometric measurement to remain consistent with a human-based morphometric measurement. However, the histogram maxima have shifted. For both biparietal and transcerebellar diameters, this systematic bias is + 2%. For the deep gray matter zone, it is about + 15%. This may be due to two (not mutually exclusive) reasons: (1) the behavior of the human expert, who may underestimate or overestimate the position of landmarks in MR images, and (2) the position of segmentation boundaries, which may be influenced by image properties. These biases can be corrected, for example by comparing the results of human experts and segmentation on a small sample of data, in order to identify and correct this bias before applying segmentation-based morphometric methods to a larger cohort. This would pave the way for the development of automated morphometric analysis, based on segmentation, which could save doctors precious time.

As regards the topological analysis of the segmentation results, the method’s connectedness score is good, with an average error of less than 1 (i.e. there is no more than one erroneous connected component per labeled region). In particular, the connectedness scores described in Fig 8 are satisfactory for 11 out of 13 regions, with two exceptions, namely the Frontal no-cingulate region and the Temporal internal region. In particular, the region with the worst connectedness score (Frontal no-cingulate) was also the region with the worst connectedness score in the Kiviat diagram.

As a result, these topological measurements can be evaluated automatically, saving doctors time and effort. For adjacency analysis, the average error is low, on the order of 0.15. More precisely, looking at the pairwise region adjacencies given in Fig 9, this error is most often equal to or very close to 0. In some cases, this error is very high, often equal to 1. This is due to the variable strength of adjacency links, which depends on the size of the interface between regions, and to the fact that the modeling of these adjacency links is currently binary. It may be further improved by (1) defining the adjacency matrix by metric rather than symbolic characterization and (2) constructing the ground truth adjacency matrix by agglomerating information from several label images. This will form part of our future work.

On reading the segmentation, clinical experts noted excellent segmentation of many volumes: the cerebellum, brainstem, corpus callosum, cingulum and temporal lobes taken as a whole. However, the experts noted variability in the demarcation line between the temporal, parietal and occipital lobes as segmented by SegSRGAN. Admittedly, these lobes are not anatomically separated by any easily discernible structure. A comparison with one or more atlases may help remove any ambiguities. As we saw earlier, some connectivity anomalies were observed in the frontal lobes, but given the overall volume of the frontal lobes, the impact on the final volume estimate is limited. The orientation of the head in the orthogonal plane has an effect on the effectiveness of SegSRGAN. Segmentation performance was considerably reduced when the head axis was far from the orthogonal plane. Indeed, as most of the clinical data followed an acquisition protocol where the orientation of the patient’s head was controlled, we did not integrate data augmentation with regard to rotations. This question could be explored further, drawing on recent work [69]. The FCOO score is easy for clinicians to use. The clinician’s delineation choices on the low resolution image are partly responsible for the error reported in the basal ganglia surface. In particular, the area behind the posterior limb of the internal capsule was delimited more restrictively by the expert than it was by SegSRGAN. In SegSRGAN, the area of the tail of the caudate nucleus was appropriately included in the deep gray matter label, which was often difficult to see in the low resolution image. The entire validation procedure described in this article enables the selection of well-segmented MR images, or some of their labels, that can be used in clinical studies. It will then be possible to correlate potential changes in regional volume with each other to identify patterns and look for correlations with outcome. If there is evidence of their relevance, these volume changes could provide early endpoints for interventional studies, accelerating the pace of research in this field.

## 6 Conclusion

In this article, we have presented new contributions relating to the analysis of premature babies’ brains from MR images. In particular, we have proposed an extended version of SegSRGAN [12], a super-resolution reconstruction and segmentation approach, which is now capable of handling multi-label segmentation instead of binary segmentation. We have also proposed a segmentation quality control protocol dedicated to the multi-criteria evaluation of multi-label segmentation results, based on morphometric and topological features. SegSRGAN and the segmentation quality control protocol have been designed for use in MRI analysis of the brain of premature infants. Nevertheless, this framework remains essentially generic. In particular, it could be adapted, modified and used for other data and clinical purposes.

We used this framework for a preliminary analysis of a subset of a large clinical cohort, namely EPIRMEX, composed of multicenter MR images. Here, our aim was to assess the ability of SegSRGAN to be applied to the whole cohort, and to identify its strengths, weaknesses and biases. The results of this study suggest the potential of SegSRGAN as a robust tool for morphometric analysis of clinical data. Further validation with multicenter data and varied resolutions is required to consolidate these results. Nevertheless, it can be further improved, for example by integrating topological information into the learning process, as studied in [70]. We can also explore the benefits of incorporating data augmentation and multi-contrast information, particularly in comparison with synthetic methods such as SynthSeg [71]. Our future work will also involve applying it more systematically to all EPIRMEX data, to enable more in-depth clinical research studies. Other applications involving the use of SegSRGAN can be envisaged. For example, we have not yet tested SegSRGAN on brains with obvious brain damage. Indeed, in the current cohort, we had too few MR images with such defects to start training the algorithm on pathological areas with dedicated labels. It would be interesting to study how SegSRGAN handles cystic leukomalacia, for example.

From a methodological point of view, we will also seek to improve / extend the proposed segmentation quality control protocol. On the topological side, based on the above discussion, we will study the coupling of topological and geometric information in the adjacency matrix, transforming it from a binary to a metric mapping. We will also seek to integrate a new module into segmentation quality control to assess the uncertainty of segmentation results.
